# Editorial: Case reports in nephrology

**DOI:** 10.3389/fneph.2025.1595978

**Published:** 2025-04-07

**Authors:** Xuefei Tian, Leopoldo Ardiles, Clay A. Block

**Affiliations:** ^1^ Section of Nephrology, Department of Internal Medicine, Yale School of Medicine, New Haven, CT, United States; ^2^ Laboratory of Nephrology, Faculty of Medicine, Universidad Austral de Chile, Valdivia, Chile; ^3^ Nephrology and Hypertension, Geisel School of Medicine at Dartmouth, Dartmouth Hitchcock Medical Center, Lebanon, NH, United States

**Keywords:** nephrology, case report, chronic kidney disease, genetic variant, infection, drug safety

## Introduction

Chronic Kidney Disease (CKD) has emerged as a critical public health challenge in the 21st century. According to the World Health Organization, approximately 850 million people globally are affected by kidney disease, with around 130 million suffering from End-Stage Kidney Disease (ESKD) requiring dialysis or kidney transplantation (1). By 2040, CKD is expected to become the fifth leading cause of reduced life expectancy worldwide. This crisis is particularly pronounced in developing countries, where 80% of ESKD patients reside, yet only 10% have access to dialysis or transplantation (2). Beyond its role in precipitating cardiovascular events and metabolic syndrome, CKD imposes a staggering socioeconomic burden, with global expenditures on kidney replacement therapy exceeding $1.5 trillion annually, far surpassing the costs for diabetes and cancer combined (3, 4).

Case reports, by their nature, tend to focus on extreme or rare conditions and would seem to be far removed from a CKD epidemic affecting millions. Osler considered it a duty for the physician to note and publish interesting cases so that similar cases could be recognized. There is now increasing recognition that investigation of the rare or extreme has additional value by generating new hypotheses and/or providing insight into common, but poorly understood pathophysiology (5, 6).

In this crucial context, the recent publication of ten case reports in “Frontiers in Nephrology” provides an essential perspective into the hidden mechanisms of rare kidney diseases and highlights the profound interconnection between clinical practice and translational research. Although we clinicians may not see these exact cases, they inspire us to be more energetic and curious when we encounter their “cousins.”

## Insights into rare and complex kidney diseases

Case reports often offer the first insights into rare kidney diseases. For instance, Kang et al. documented a case of polycystic kidney disease (PCKD) complicated by xanthogranulomatous pyelonephritis (XGP), highlighting the potential risk of cysts as infection foci. Despite the absence of a history of urinary tract infection or stones, cysts can lead to urine stasis and bacterial colonization, resulting in granulomatous inflammation. This report suggests that structural kidney diseases such as PCKD and duplicated kidneys might be hidden etiological factors, a mechanism consistent with that proposed by Jang et al. as the “chronic obstruction-immune dysregulation” model (7). This underscores the necessity for enhanced imaging surveillance in patients with structural kidney diseases.

## Diagnostic challenges and therapeutic interventions

Kidney diseases often present with non-specific symptoms, making diagnosis challenging. Zhang et al. reported three cases of emphysematous pyelonephritis (EPN), where one patient died due to treatment cessation by the family, while the other two were successfully treated surgically. All patients had diabetes with poor glycemic control, and the primary pathogen was *E. coli*. The authors emphasized the importance of CT diagnosis, antibiotic therapy, glycemic control, and timely surgical intervention. Zhang et al. provided a practical framework for EPN management, stressing CT-guided classification, early drainage, and nephrectomy of non-salvageable kidneys. However, this series also highlighted systemic challenges: small-scale studies hindering protocol standardization, speculative biomarker utility, and socio-cultural influences on outcomes. Future research should prioritize multi-center cohorts to improve risk scoring, evaluate laparoscopic versus open surgery, and explore the prognostic application of biomarkers like IL-6. Additionally, educating patients and families on the severity of EPN could mitigate premature treatment discontinuation.

## Advanced diagnostics and multidisciplinary approaches

Leiva et al. highlighted the complexities in managing pregnancy-associated thrombotic thrombocytopenic purpura (TTP) in systemic lupus erythematosus (SLE) patients. With ADAMTS13 activity at just 6% and a “full-house” immunofluorescence pattern on kidney biopsy, the case underscored the intricate interplay between SLE and thrombotic microangiopathy (TMA). This case supports the 2020 International Consensus on TMA (8) and calls for developing pregnancy-safe TTP therapies. The patient’s progression to ESKD highlights the chronic kidney damage mechanisms in SLE-associated TMA, necessitating lifelong nephrological monitoring.

Burbano et al. reported a fatal nephrobronchial fistula resulting from xanthogranulomatous pyelonephritis, emphasizing the aggressive nature of advanced XGP involving visceral fistulas. Utilizing enhanced CT and pathological correlations, the study stressed the need for multidisciplinary collaboration in managing complex infections. Despite adherence to standard treatment protocols (antibiotics and nephrectomy), the patient succumbed to multi-organ failure post-surgery. This case aligns with historical findings on XGP’s aggressive nature, particularly with visceral fistulas, and suggests anatomical or pathophysiological differences worthy of further investigation.

Errabelli et al. presented a compelling case of pseudo-acute kidney injury (AKI) induced by the CDK4/6 inhibitor abemaciclib. This case highlighted the superior diagnostic capability of cystatin C in identifying renal tubular secretion dysfunction. Their findings directly support the “biomarker stratified diagnostic process” proposed by Vanhoutte et al. (9). They recommend prioritizing cystatin C over serum creatinine to assess kidney function in patients using renal tubular secretion inhibitors.

## Genetic insights and personalized medicine

Case reports provide critical evidence for genotype-phenotype correlations in hereditary kidney diseases. Krall et al. identified an *NPHS1* mutation (p.R711S) in the Māori population, presenting a milder disease course compared to the Finnish-type congenital nephrotic syndrome (CNS). This observation suggests that genetic background may influence disease trajectories by retaining some nephrin function or activating compensatory pathways, offering new insights for *NPHS1* genotype-phenotype correlations and gene therapy (10).

Paladugu and Vukkadala discussed compound heterozygous *SLC2A9* variants in South Asian patients with Renal Hypouricemia Type 2 (RHUC2), highlighting the genetic diversity within this group. Integrating whole genome sequencing (WGS) with clinical phenotyping, the study validated the pivotal role of genetic testing in atypical presentations such as low-intensity exercise-induced acute kidney injury (AKI), in alignment with guidelines by Nakayama et al (11). The management strategy emphasized conservative treatment efficacy and called for multi-center clinical trials to evaluate the potential of xanthine oxidase inhibitors in RHUC2. By openly sharing genomic data, the authors promoted global collaborative research on rare kidney diseases.

Ignacio Alarcón et al. reported a novel *ACTN4* gene mutation (c.625_633del), contributing new data to the genetic basis of steroid-resistant nephrotic syndrome. The authors utilized a comprehensive diagnostic approach, integrating clinical history, laboratory tests, imaging studies, kidney biopsy, and whole-exome sequencing. Initially classified as a “variant of uncertain significance” (VUS), the mutation was eventually reclassified as “likely pathogenic.” Although there was no recurrence five months post-transplantation, the lack of long-term follow-up data and functional validation experiments highlighted the need for more robust follow-up systems and detailed molecular mechanism studies in hereditary kidney disease research.

## Medication safety and toxicity

Case reports play a pivotal role in drug safety monitoring. Zhang et al. detailed a roxadustat overdose, revealing significant short-term hemoglobin elevation followed by a stealthy rise in serum creatinine over nine months, indicating potential long-term risks. This case enhances toxicological literature on HIF-PHI, underscoring the need for vigilance even with seemingly “safe” overdoses, balancing therapeutic efficacy with potential uncertainties.

Alamilla-Sanchez et al. reported on platinum-induced distal tubular damage, challenging the traditional view of proximal tubular nephrotoxicity predominance. Using urinary calcium-creatinine ratio and fractional excretion of magnesium, the study distinguished Bartter-like from Gitelman-like phenotypes, suggesting a genetic susceptibility hypothesis for chemotherapy-induced nephrotoxicity. This aligns with Nozu et al.’s hereditary tubulopathies classification (12), indicating the need for routine distal tubular function monitoring in chemotherapy patients.

## Future directions and broader implications

While case reports are clinically significant, their limitations are apparent. Case reports’ inherent evidence level (CEBM Level 4) makes establishing causality challenging. Many case reports remain primarily observational, relying on subsequent studies for mechanism elucidation. Translational research depends on deep collaboration between clinicians and basic scientists, exemplifying “bedside-to-bench” team models. Emerging technologies like patient-specific organoids enable the direct validation of drug toxicity mechanisms *in vitro* (13).

## Conclusion

Case reports embody the synthesis of medical humanities and scientific rigor. From precise categorization of pregnancy-associated TTP to expanding etiologies of PCKD complicated by XGP, and from redefining platinum nephrotoxicity mechanisms to delineating population-specific genetic variants, these reports are not merely academic annotations but engines of scientific advancement. They document clinical practice’s puzzles and epiphanies, illustrating the interweaving of individual destinies with medical inquiry. Each meticulously documented case may unlock secrets that ultimately redefine medical textbooks. The wisdom garnered from case reports will ultimately catalyze changes in clinical practice. Through this continued accumulation, case reports will harness the necessary force to transform patient care, driven by scientific curiosity and human warmth, paving the way for new horizons in nephrology.
